# Valorization of Dairy By-Products, Sweet Whey, and Acid Whey, in the Production of Fermented Black Carrot Juice: A Comparative Study of the Phytochemical, Physicochemical, Microbiological, and Sensorial Aspects

**DOI:** 10.3390/foods14020218

**Published:** 2025-01-12

**Authors:** Hacer Çoklar, Mehmet Akbulut, Ali Aygun, Muhammed Talha Akbulut

**Affiliations:** 1 Department of Food Engineering, Agriculture Faculty, Selcuk University, Konya 42130, Türkiye; hacercoklar@selcuk.edu.tr; 2 Department of Animal Science, Agriculture Faculty, Selcuk University, Konya 42130, Türkiye; aaygun@selcuk.edu.tr; 3 Department of Food Engineering, Agriculture Faculty, Tekirdağ Namık Kemal University, Tekirdağ 59030, Türkiye; talhaakbulut@nku.edu.tr

**Keywords:** anthocyanins, anthocyanin-protein complex, color, fermentation, hierarchical cluster analysis, lactic acid bacteria, lactic beverage, organic acids, principal component analysis, yeast

## Abstract

The aims of this study were to improve the functional and nutritional properties of fermented black carrot juice by using sweet and acid whey in the production of fermented black carrot juice, to transform whey into a value-added product and to determine the effect of whey addition on the fermentation process. Whey was utilized as a water substitute in the formulation of the beverage prior to fermentation, and five distinct formulations were developed based on the type and proportion of whey (0% whey (control sample), 25% acid whey, 100% acid whey, 25% sweet whey, 100% sweet whey). Microbiological, sensorial, phytochemical, and physicochemical analyses were performed on samples taken during fermentation and on samples fermented and then resting. The addition of whey into the formulation resulted in an increase in acidity and turbidity of the beverage, with lower anthocyanin content observed in samples containing whey compared to the control throughout the fermentation process. The samples containing 100% whey exhibited lower a*, b*, *h*, and C* values and lower amounts of individual anthocyanins. The microbial load in these samples was high in the early stages of fermentation and reached a minimum towards the end of fermentation. The incorporation of whey led to an acceleration in the fermentation process, an enhancement in the microbiological characteristics of the beverage, and a substantial variation in phenolic compounds through the formation of a reversible protein complex. The resting process provided significant increases in color, anthocyanins, and gentisic and chlorogenic acids of whey-containing samples. The results showed that it is possible to produce whey-based functional fermented black carrot juice that is close to the control sample in terms of sensory and phytochemical properties and better than the control sample in terms of lactic acid bacteria count. It is recommended that both sweet and acid whey be utilized at a ratio of 25% in the production of fermented black carrot juice and to rest at 4 °C before consumption.

## 1. Introduction

Whey can be defined as the liquid that remains after milk coagulates and the curds are separated. It is a by-product of the dairy industry and can be divided into two different types: sweet and sour. Sweet whey, also known as cheese whey, is produced in the cheese making process by coagulating milk with rennet and is characterized by a pH of 5.6 or higher. In contrast, acid whey, also known as yogurt whey, is produced by the fermentation of lactose to lactic acid and has a pH between 3.6 and 4.5 [1]. It is estimated that approximately 180–190 million tons of waste whey, 2.8 million tons of condensed whey, and 3.2 million tons of dried whey are produced annually worldwide [2,3]. The water content of sweet and acid whey ranges from 93 to 94% and 94 to 95%, the total solids content from 6.0 to 6.5% and 5 to 6%, and lactose content from 4.5 to 5.0% and 3.8 to 4.3%. The total protein, whey protein, citric acid, and ash (mineral) contents are approximately the same in both types of whey, ranging from 0.8% to 1.0%, 0.60% to 0.65%, and 0.5% to 0.7%, respectively. Sweet whey contains trace amounts of lactic acid, while acid whey may contain up to 0.8% lactic acid [4]. The primary protein component of whey is β-lactoglobulin, which represents 45–58% of the total protein content. Other whey proteins are α-lactalbumin (13–25%), glycomacropeptides (10–20%), bovine serum albumin (6–12%), immunoglobulins (9–14%), lactoferrin (1–2%), lactoperoxidase (0.5%), and lysozyme (0.0003%) [5]. Whey proteins are used in the formulation of many products such as bakery and confectionery products, baby food, yogurt, ice cream, and thirst-quenching drinks due to their nutritional and health-promoting and functional properties such as essential amino acids, immunomodulatory, anticarcinogenic, antiviral, antimicrobial, antioxidant, tumor inhibitory, and growth inhibitory effect on human breast cancer cells [5,6,7]. Research has been conducted on the use of whey in lactic beverages, considering that it can accelerate fermentation as an important substrate that microorganisms can use in addition to its nutritional properties, and beverages using whey in a mixture with milk, fruit juice and probiotic bacteria, and yeasts have been developed [8,9,10].

Fermented black carrot juice is a sour, cloudy, red-colored beverage produced by the spontaneous lactic acid fermentation of black carrots, turnips, sourdough, bulgur flour, salt, and water. The dark red color of the beverage is due to the anthocyanins of black carrots, which are used as the main raw material in its production [11]. The primary anthocyanin compounds present in black carrots are cyanidin glycosides. Anthocyanins are important for both quality and health. Previous studies have shown that significant changes in anthocyanins occur during lactic acid and alcohol fermentation [12,13]. Acetaldehyde produced during yeast fermentation and ethanol oxidation can lead to the polymerization of polyphenols [14]. The reaction of anthocyanins with acetaldehyde results in the formation of anthocyanin oligomers, which leads to changes in color and sensory properties of the product [15]. Proanthocyanidins are phenolics with low stability, and they can undergo different reactions depending on the environmental conditions. These reactions result in the formation of colorless compounds or intensely colored anthocyanins with increased stability [16]. Polyphenolic compounds have the ability to bind with proteins, resulting in the formation of sediment [17]. The complexation of anthocyanins with proteins reduces their discoloration and oxidative degradation and increases their thermal stability, providing better protection against degradation during the intestinal digestion process [18].

Considering the nutritional composition of whey, especially its protein content, it is extremely important to offer it to the consumer in the composition of different alternative products, both for the increased and continuous evaluation of whey and for the enrichment of different foods. In recent years, whey has been incorporated into beverage formulations for the purpose of both waste utilization and nutritional enrichment. In previous studies, an unfermented beverage containing jabuticaba peel anthocyanins and whey protein [19], a whey–blackberry juice blend beverage fermented with lactic acid bacteria [20], a carrot juice whey-enriched beverage fermented with milk or water kefir [21], a fermented dairy beverage enriched with whey and blueberry [22], a functional dairy milk beverage containing whey fermented with *Lactobacillus paracasei* [23], a blueberry juice beverage with whey fermented with *Lactobacillus plantarum* [24], a fermented beverage containing mulberry and whey [10], a beverage fermented with *Lactobacillus plantarum* or *Lactobacillus casei* containing whey protein and blueberry juice [25], and apple, pear, blueberry, and strawberry beverages with whey [26] have been prepared and the effect of whey addition on the quality parameters of the obtained beverages has been tried to reveal.

In a previous study, Güven, N. et al. [27] used whey in the production of fermented black carrot juice with the aim of reducing salt content. However, this study differs from the previous study in terms of both the formulation and the product evaluation. In this study, two different types of whey were incorporated into the formulation at concentrations of 0%, 25%, and 100%.

The objectives of this study were to use sweet and acid whey in the production of fermented black carrot juice to improve its functional properties, thereby converting whey into a high value-added product, and to determine the effect of whey addition on the fermentation process of fermented black carrot juice and the physicochemical, phytochemical, and sensory properties of the final product. The study also aimed to identify changes that occur during resting and to determine similarities by grouping fermented black carrot juices using chemometric methods.

## 2. Materials and Methods

### 2.1. Material

The bulgur flour, salt, water, *Saccharomyces cerevisiae*, and black carrot used in the production of fermented black carrot juice in this study were purchased from a local market. The sweet whey (SW) and acid whey (AW) were obtained from Enka Milk and Dairy Products, a company engaged in the production of cheese and strained yogurt in Konya. Both whey samples were unpasteurized and quickly transported to the laboratory after their production. The protein ratio of the whey used was 2.08% in AW and 0.42% in SW, the fat ratio was 0.065% in AW and 0.060% in SW, the sugar ratio was 3.58% in AW and 4.92% in SW, and the salt ratio was 0.20% in AW and 0.24% in SW.

### 2.2. Fermentation Method

Fermented black carrot juices were prepared using a two-step fermentation method. First, bulgur (0.91%) and yeast (0.2%) were fermented in a glass jar (10 L) at room temperature for 1 day. Then, salt (1.16%) and black carrot (16.6%) were added to the jar, and it was filled with water to 10 L. The sample prepared according to this formulation was used as a control. Sweet and acid whey were used as water substitutes in the formulation. The first formulation consisted of 25% sweet whey and 75% water, the second formulation consisted of 100% sweet whey and 0% water, the third formulation consisted of 25% acid whey and 75% water, and the fourth formulation consisted of 100% acid whey and 0% water. The jars were capped and left to ferment at 25 °C. The samples were coded as SW-25, SW-100, AW-25, and AW-100 according to whey content and whey type, respectively. The progress of fermentation was monitored by measuring the total titration acidity and was terminated when the increase in acidity ceased. Lactic acid bacteria count, total acidity, pH, color, turbidity, total monomeric anthocyanin content, and phenolic profile analysis were performed on samples taken on the 20th and 40th days of fermentation to evaluate the whey type and ratio of the fermentation period, and sensory analysis, organic acid profile, ethanol content, total acidity, pH, turbidity, phenolic profile, and color analysis were performed to determine the effect of resting on samples made ready for consumption and rested at +4 °C.

### 2.3. Determination of Titratable Acidity, pH, Turbidity, and Color Parameters

The pH values of the samples were determined by direct measurement with the pH meter (Inolab 720, WTW, Weilheim, Germany). To determine the acidity, the samples were titrated with NaOH solution (0.1 N) until the pH reached 8.1 and the results were expressed as g lactic acid (LA) equivalents/100 mL. The turbidity was measured with a turbidimeter (WTW TURB 430 T) and the results were reported as Nephelometric Turbidity Units (NTUs). The CIELAB color values (L*, a*, b*, C*, and *h*) of the samples were measured with the spectrophotometer (CM-5, Konica Minolta, Osaka, Japan) equipped with the cell holder (10 mm, CM-A207) [12].

### 2.4. Determination of Total Monomeric Anthocyanin Content

The total monomeric anthocyanin content in the fermented black carrot juice was determined by the pH difference method described previously [28]. Briefly, 1 mL of sample was transferred to two separate tubes. The first tube was diluted with 4 mL of pH 1.0 buffer (potassium chloride, 0.025 M) and the second tube was diluted separately with pH 4.5 buffer (sodium acetate, 0.4 M). After 30 min, the wavelengths at 515 and 700 nm were measured with a spectrophotometer (U-1800, Hitachi, Japan), and the difference in absorbance was calculated according to Equations (1) and (2):

A = (A_520nm_ − A_700nm_) _pH1.0_ − (A_520nm_ − A_700nm_)_pH4.5_ (1)

Total monomeric anthocyanins (TMA) (mg CGE/L) = (A × MW × DF × 1000)/(ε × l)(2)

where MW (molecular weight) = 449.2 g/mol for cyanidin- 3- glucoside (CG); ε = 26,900 M extinction; coefficient, in L × mol^−1^ × cm^−1^; l = path length in cm; DF = dilution factor.

### 2.5. Determination of Individual Phenolic Compounds

The phenolic compounds of fermented black carrot juice were purified using C18 solid phase extraction (SPE) cartridges (Agilent, Santa Clara, CA, USA). After loading the sample into the cartridges, the phenolics and anthocyanins were eluted with ethyl acetate and methanol. The eluates were evaporated at 35 °C, resuspended in 1 mL of methanol, and then filtered through a 0.45 μm pore size syringe filter (Sartorius AG, Göttingen, Germany). Analysis of the phenolic compounds in the samples was performed on an Agilent 1260 Infinity Series HPLC system, which was equipped with a diode array detector. The separation was performed on a reverse-phase C18 column (5 μm, 250 mm × 4.6 mm i.d.). The mobile phase consisted of a solution of acetic acid in water and a solution of water, acetonitrile, and acetic acid. The flow rate was 0.75 mL/min. The detector was set at 280, 320, and 360 nm for the phenolics and 520 nm for the anthocyanins.

### 2.6. Determination of Organic Acids and Ethanol Content

Fermented black carrot juice was diluted 1:2 (*v*/*v*) with ultrapure water and passed through a polyvinylidene fluoride (PVDF) filter with a pore diameter of 0.45 μm and analyzed by HPLC (1260 Infinity Series, Agilent, Baden-Württemberg, Germany). Separation of organic acids and ethanol was performed on an Aminex HPX-87H column. Sulfuric acid solution (0.005 N) was used as the mobile phase at a flow rate of 0.6 mL/min, organic acids were detected by a DAD detector set at 210 nm, and ethanol was detected by a refractive index detector [28].

### 2.7. Determination of Lactic Acid Bacteria (LAB) and Yeast Counts

One ml of the samples was taken and homogenized with 9 mL of sterile peptone water, and serial dilutions of 10⁻^1^ to 10⁻⁸ were prepared. One ml of the diluted sample was transferred to separate Petri dishes for the enumeration of lactic acid bacteria (LAB) and yeast. In addition, deMan Rogosa Sharpe (MRS) agar was used for the enumeration of LAB, while potato dextrose agar was used for the enumeration of yeast. Petri dishes were incubated at 28 °C for 4 days under aerobic conditions for yeast enumeration, and MRS agar was incubated at 35 °C for 72 h under anaerobic conditions [29].

### 2.8. Sensorial Evaluation

The sensory evaluation of fermented black carrot juices was conducted by a panel of 20 semi-trained males and females aged 25–50 years. The panelists were provided with 10 mL of the samples, accompanied by whole grain wheat crackers and water, in 20 mL glasses at a temperature of 5 °C. They were then asked to rate the samples on a 9-point hedonic scale (9 = extremely liked; 1 = very disliked) for color, aroma, taste, appearance, and overall acceptability [29].

### 2.9. Statistical Analysis

The results are presented as mean ± standard deviation. To determine whether fermentation time and sample type affect the quality parameters of fermented black carrot juices and whether there is a difference between the samples after resting at 4 °C at the end of fermentation, variance analysis was applied to the data. Tukey’s comparison test was applied to the identified sources of variation that were found to be significant in the analysis of variance. To determine the effect of resting, a two-sample t-test was performed on the data obtained on the 40th day of fermentation samples and the samples that were rested at 4 °C. In addition, both fermentation time and resting samples were grouped using principal component and hierarchical clustering tests, which revealed similarities among the samples. Principal component analysis (PCA) and hierarchical cluster analysis (HCA) were used as chemometric methods to reduce the number of variables based on their similarities. Statistical analyses were performed using MINITAB 19 (Minitab Inc., State College, PA, USA).

## 3. Results

### 3.1. Physicochemical and Phytochemical Properties of Samples

This research was carried out by monitoring the progress of fermentation through analyses performed on the 20th and 40th days of fermentation and then evaluating the analyses performed after cold storage at 4 °C to be ready for consumption.

The pH, titratable acidity, and turbidity values of the fermented black carrot juice samples obtained during fermentation are presented in Table 1.

The pH value and titration acidity of the samples varied according to the whey type and ratio, but it was found that there was no statistically significant change in pH and acidity as the fermentation progressed. The highest pH (3.49) and the lowest acidity (0.349%) were determined in the control sample on the 20th day of fermentation. When both stages of fermentation were considered, the order of the samples by pH was as follows: control > AW-100 > SW-100 > AW-25 > SW-25. It was observed that the addition of whey to the formulation increased the acidity of the samples and at the same time, as the ratio increased, the acidity also increased. On the 20th day of fermentation, the acidity ranking of the samples was as follows: control < AW-25 < SW-25 < SW-100 < AW-100.

The turbidity of the beverages varied both with fermentation period and sample type (*p* < 0.01). It was found that the addition of whey increased the turbidity, especially the beverages containing acid whey, which were more turbid than the others, and the turbidity of all the samples decreased with the progress of fermentation. On the 20th day of fermentation, the turbidity of all the samples was higher than that of the control (188.50 ± 6.50), while on the 40th day of fermentation, the turbidity of the samples containing 25% whey was lower than that of both the control and the samples containing 100% whey. The total monomeric anthocyanin amounts of fermented black carrot juices varied with the type and proportion of whey and were not affected by the fermentation process. The anthocyanin amount of the control sample was determined to be 63.62 mg/L on the 20th day and 68.61 mg/L on the 40th day of fermentation, showing a higher concentration than that of all other samples. The lowest anthocyanin amounts were observed in samples containing 100% sweet or acid whey, while the anthocyanin amounts increased as the whey ratio decreased.

The pH, acidity, and turbidity values of the samples whose fermentation was completed and rested at 4 °C before consumption are presented in Table 2. The highest pH value was observed in the control sample, with a value of 3.46, followed by AW-100, SW-100, AW-25, and SW-25, in decreasing order. The pH values of the AW-25 and SW-25 samples were found to be statistically similar. The lowest acidity value was observed in the control sample (0.490 ± 0.020). It was observed that the acidity of the formulations containing 100% whey was the highest, while the acidity of the formulations containing 25% whey was lower. Turbidity values at the resting stage ranged from 54.05 to 71.60 NTUs, and no statistically significant difference was observed.

The two-sample *t*-test was used to determine whether the pH, acidity, and turbidity values of the samples taken on the 40th day of fermentation, changed after resting for 2 days at 4 °C (Table S1). The change in the turbidity of the fermented black carrot juice containing 100% acid whey after resting was found to be significant (*p* < 0.05), while the turbidity, acidity, and pH values of the other samples did not change with resting.

The control sample had the highest anthocyanin content among the samples that were stored at 4 °C, followed by the SW-25 sample. It was observed that the anthocyanin content of the SW-100, AW-25, and AW-100 samples was lower than those of the SW-25 and control samples, and statistically equal to each other. When the samples taken on the 40th day of fermentation were compared with the resting samples (Table S1), it was found that the amount of anthocyanin was different and higher in all the samples except the control sample.

The reflectance color parameters (L*, a*, b*, C*, and *h*) of fermented black carrot juices were found to be affected by both the progress of fermentation and the type and ratio of whey that was added to the formulation (Table 3). On the 20th day of fermentation, the L* value, which represents brightness, did not differ significantly from that of the samples containing 100% whey, but was observed to be higher than that of the samples containing 25% whey. As fermentation progressed, the brightness of all samples increased. The highest value was observed in AW-100, while the lowest value was observed in SW-100. On the 20th day of fermentation, the highest a* (redness) and b* (yellowness) values were observed in the control sample. For these two-color parameters, the control was followed by samples containing acid whey, and the lowest value was observed in the SW-100 sample. As the fermentation progressed, the redness value of the samples increased, resulting in the following order: AW-25 > SW-25 > control > AW-100 > SW-100, the redness value of the samples increased, resulting in the following order: AW-25 > SW-25 > control > AW-100 > SW-100. For the yellowness value (b*), there was an increase in the control, SW-25 and AW-25 samples as the fermentation progressed, a* decrease in the AW-100 samples and there was no change in the b* value of the SW-100 sample. It was determined that the C* value (representing the saturation) of the control was the highest at the first stage of fermentation, and the saturations of the samples containing acid whey were almost equal and more saturated than those of the samples containing sweet whey. On the 40th day of fermentation, it was observed that the samples containing 25% whey had higher saturation values than the control sample, although the difference was not statistically significant. The saturation values of the samples containing 100% sweet and acid whey were found to be lower than in all other samples. The *h* value of all the samples, except for the AW-100, increased with the progress of fermentation. The highest and lowest values of *h* were found in the samples SW-25 and AW-100, respectively, on the 40th day of fermentation.

**Figure 1:**
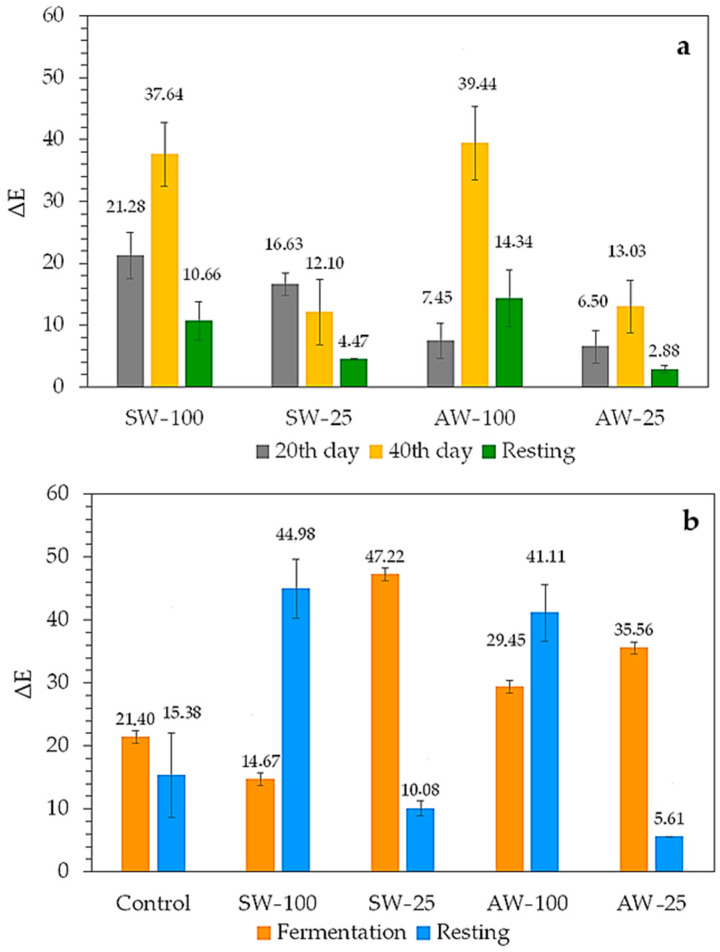
ΔE values of fermented black carrot juice samples. (**a**) Color changes in whey-containing samples compared to control on 20th and 40th days of fermentation and after resting. (**b**) Color changes in all fermented black carrot juices during fermentation and after resting. For the ΔE values in fermentation, which are represented by the blue column, the data from the 20th day of each sample were selected as L*_0_, a*_0_, and b*_0_. Similarly, for the ΔE values in rest, which are shown in the red column, the values from the 40th day of each sample were chosen as L*_0_, a*_0_, and b*_0_. (SW-25: 25% sweet whey, SW-100: 100% sweet whey, AW-25: 25% acid whey, AW-100: 100% acid whey).

The color values of fermented black carrot juices appear to be significantly affected by both the type and proportion of whey used and the fermentation time. To facilitate a more detailed examination of this phenomenon, ΔE values representing the color difference are presented in Figure 1a,b. The ΔE value is used to determine the degree of similarity in color between the control and the processed sample, with a higher value indicating a lower degree of similarity. Figure 1a illustrates the ΔE values determined in samples containing whey during fermentation and after resting at 4 °C. The color parameters of the control, which is fermented black carrot juice without whey, were used to determine the ΔE value.

On the 20th day of fermentation, it can be seen that the color of the AW-25 was closer to the control, and on the 40th day of fermentation, both SW-25 and AW-25 samples were closer to the control sample in terms of color. On the 40th day of fermentation, samples containing 100% sweet whey and 100% acid whey showed significant differences in color compared to the control sample. When the samples rested at 4 °C for 2 days were examined, it was found that the samples containing 25% whey and especially AW-25 were closer to the control sample and that all the samples were more similar to the control sample in terms of color. It can be concluded that resting at 4 °C had a positive effect on the color of the samples containing whey. Figure 1b shows the color difference in the samples between the 20th and 40th days of fermentation and after resting at 4 °C. For the determination of ΔE, each sample was evaluated individually, and the values of the 20th and 40th days of fermentation were used in the calculation of the columns shown in blue, and the color values of the samples on the 40th day of fermentation and after resting at 4 °C were used in the calculation of the columns shown in red. The greatest color difference during fermentation was observed in the SW-25 sample, followed by the AW-25 sample. At rest, the greatest difference was in the SW-100 sample, followed by the AW-100 sample. The least difference in color at rest was seen in the AW-25 and then in the SW-25 samples. In summary, the samples with the greatest color difference as fermentation progressed were those containing 25% whey, and the samples with the greatest color difference after resting were found to be those containing 100% whey.

Table 4 shows the color values of fermented black carrot juices rested at 4 °C for 2 days. The L* values of the samples varied between 34.19 and 40.05, a* values between 56.89 and 64.33, b* values between 41.56 and 52.81, C* values between 72.98 and 81.77, and *h* values between 34.71 and 40.23. It was observed that there were no statistically significant differences present between the samples. The results of the two-sample t-test performed on the 40th day of fermentation samples and the samples rested at 4 °C for two days are presented in Table S1. No statistically significant differences were observed in the color parameters of the samples.

Gentisic, chlorogenic, caffeic, *p*-coumaric and ferulic acids, cyanidin-3-xylosylgalactoside, cyanidin-3-xylosylglucosylgalactoside, and sinapic, ferulic, and *p*-coumaric acid derivatives of cyanidin-3-xylosylglucosylgalactoside were detected in fermented black carrot juice. The fermentation period had no statistically significant effect on the phenolic acids present in the fermented black carrot juice, except for *p*-coumaric acid, but whey addition and ratio were effective (Table 5). The highest gentisic acid was detected in the control and the amount of gentisic acid decreased as the whey ratio increased. Gentisic acid was detected at similar levels in AW-25 and AW-100. The highest amounts of chlorogenic, caffeic, and *p*-coumaric acids were detected in AW-100. While ferulic acid was not detected in the control, the highest amount of ferulic acid was found in SW-25. The fermented black carrot juices rested at 4 °C showed differences in the amounts of gentisic and chlorogenic acids, but the difference in the amounts of caffeic, *p*-coumaric, and ferulic acids was found to be statistically insignificant (Table 6).

The highest amounts of gentisic acid and chlorogenic acid were found in the control sample, followed by SW-100, AW-25, SW-25, and AW-100 in descending order. However, the amounts of gentisic acid and chlorogenic acid in the control and SW-100 samples were not statistically different from each other. It was observed that the samples containing sweet whey had chlorogenic acid content that was almost identical to that of the control sample and the samples containing acid whey. It was found that the ratio of whey had no effect on the concentration of chlorogenic acid. The lowest levels of caffeic and *p*-coumaric acids were observed in AW-100. Although not statistically significant, the amount of ferulic acid rested fermented black carrot juices increased as the whey ratio increased.

A statistically significant difference was observed in the gentisic acid and chlorogenic acid content of SW-100 and in the chlorogenic acid content of AW-100 between the unrested samples and the samples rested at 4 °C. After the resting, there was an increase in the gentisic and chlorogenic acid content of SW-100 and a decrease in the chlorogenic acid content of AW-100. The difference between the other phenolic acid contents of the samples taken on the 40th day of fermentation and samples rested in cold afterwards was found to be statistically insignificant (Table S2).

The amounts of all the anthocyanins in fermented black carrot juices varied with the addition of whey but were not affected by the fermentation process. The amount of cyanidin-3-xylosylglucosylgalactoside was higher in SW-25 and AW 25 than in the control and lower in SW-100 and AW-100 than in the control (Table 7). The fermented black carrot containing sweet whey juice had more cyanidin-3-xylosylglucosylgalactoside than the samples containing acid whey. The highest concentration of cyanidin-3-xylosylgalactoside was observed in the control sample, with a value approximately 10 times that of the SW-100 sample, 25 times that of the AW-100 sample, 1.5 times that of the SW-25 sample, and 2.5 times that of the AW-25 sample. While similar amounts of cyanidin-3-xylosyl(cinnapolyglucosyl)galactoside were detected in the control, SW-25, and AW-25 samples, SW-100 contained one quarter of this anthocyanin detected in these three samples, and AW-100 contained one eighth. According to the amount of cyanidin-3-xylosyl(feruloylglucosyl)galactoside and cyanidin-3-xylosyl(coumaroylglucosyl)galactoside, the samples were ranked as control > SW-25 > AW-25 > SW-100 > AW-100. The amounts of cyanidin-3-xylosylglucosylgalactoside, as well as cyanidin-3-xylosyl(feruloylglucosyl)galactoside and cyanidin-3-xylosyl(coumaroylglucosyl)galactoside, were higher in samples containing sweet whey. The lowest levels of all individual anthocyanins were found in AW-100 and then in SW-100. After resting, no statistically significant difference was observed among the samples in terms of cyanidin-3-xylosylglucosylgalactoside amounts. According to the amounts of cyanidin-3-xylosylgalactoside and cyanidin-3-xylosyl(feruloylglucosyl)galactoside, the samples were ranked in a decreasing order as control, SW-100, SW-25, AW-100, and AW-25. However, the difference between the control and SW-100 samples was not statistically significant. The amount of cyanidin-3-xylosyl(sinapolyglucosyl)galactoside in all the samples with added whey was almost the same and lower than in the control sample.

The amount of cyanidin-3-xylosyl(coumaroylglucosyl)galactoside was the highest in SW-100 after resting. This was followed by the control, AW-100, SW-25, and AW-25 in a decreasing order. However, the amounts of cyanidin-3-xylosyl(coumaroylglucosyl)galactoside in samples SW-25, AW-25, and AW-100 did not show statistically significant differences. When the samples of the 40th day of fermentation were compared with the samples rested at 4 °C, it was observed that the individual anthocyanins of the samples containing 25% whey did not differ according to the resting. The amount of cyanidin-3-xylosyl (feruloylglucosyl) galactoside in the control sample, the amounts of cyanidin-3-xylosylglucosylgalactoside, cyanidin-3-xylosylgalactoside, and cyanidin-3-xylosyl (feruloylglucosyl) galactoside in the SW-100 sample, and the amounts of cyanidin-3-xylosylgalactoside and cyanidin-3-xylosyl (feruloylglucosyl) galactoside in the AW-100 sample were statistically different (Table 8). It can be concluded that resting has an increasing effect on some anthocyanins of the samples containing 100% whey.

The organic acid profile and ethanol analyses were performed on samples rested at 4 °C for two days. The results are shown in Table 9. Lactic, formic, propionic, and acetic acids were detected in the control. In addition to these organic acids, citric acid was detected in all whey-containing samples. Succinic acid was detected in all samples, but below the limit of detection. The amounts of lactic acid, acetic acid, and formic acid in the control sample were found to be lower than in the fermented black carrot juices containing whey. The highest amount of citric acid was found in AW-100 and the lowest in AW-25. Higher amounts of lactic acid were determined in samples containing 100% whey, and the lactic acid content of samples containing acid whey was higher than that of samples containing sweet whey and the control. The order of the samples in a descending order of propionic acid content was as follows: AW-100, control, AW-25, SW-100, and SW-25. The formic acid content of the samples containing 100% whey was found to be higher than that of the samples containing 25% whey. The control sample had the lowest ethanol content, followed by SW-25, SW-100, AW-25, and AW-100 in ascending order. The AW-100 sample had the highest concentration of all organic acids and ethanol.

### 3.2. Microbiological Properties of Fermented Black Carrot Juice Samples

The lactic acid bacteria count of fermented black carrot juice was affected by both the progress of fermentation and the proportion and type of whey added to the formulation (Table 10). On the 20th day of fermentation, the lactic acid bacteria count in the fermented black carrot juice that contained 100% whey was found to be higher than that of the control and the samples that contained 25% whey. On the 40th day of fermentation, the samples were ranked by the number of lactic acid bacteria in the following order: SW-100 < AW-100 < Control < AW-25 < SW-25. The yeast count of fermented black carrot juices was not influenced by the addition of whey but was influenced by the fermentation process. On the 20th day of fermentation, the yeast count of the samples varied between 6.70 and 8.11 log CFU/mL. Although no statistical difference was found, the samples were ranked as control < SW-25 < AW-25 < SW-100 < AW-100 on the 20th day of fermentation. On the 40th day of fermentation, the yeast count showed a significant decrease in all samples and varied between 4.07 and 4.44 log CFU/mL. Overall, the microbial load in samples containing 100% whey was high during the initial stages of fermentation and reached a minimum on the 40th day of fermentation.

### 3.3. Sensorial Properties of Fermented Black Carrot Juice Samples

**Figure 2:**
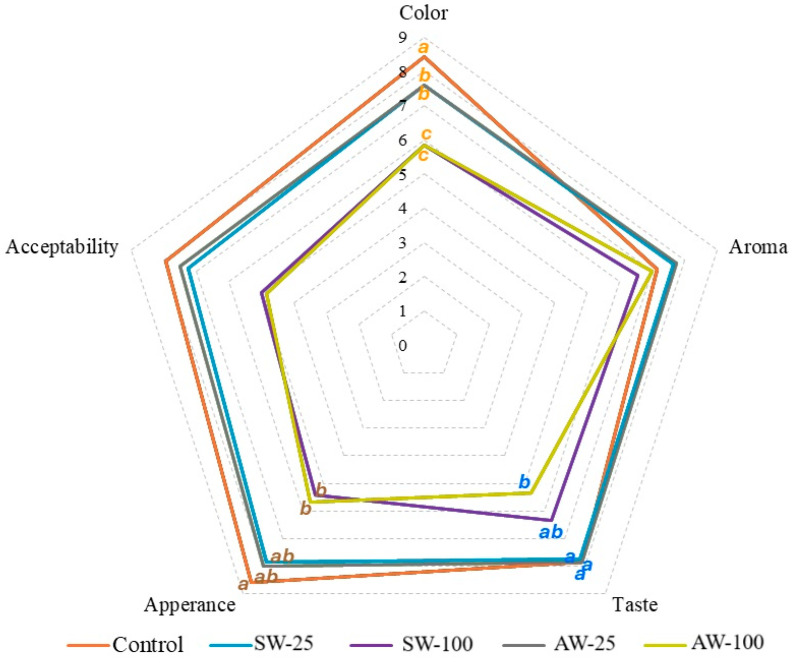
The radar plot of sensory evaluation of fermented black carrot juice samples (SW-25: 25% sweet whey; SW-100: 100% sweet whey; AW-25: 25% acid whey; AW-100: 100% acid whey). SW—sweet whey; AW—acid whey; different letters in the same color indicate the difference between means. Numbers between 0–9 show the degree of liking (9 = extremely liked; 1 = very disliked).

In the sensory evaluation of the fermented black carrot juice, the panelists were requested to score the samples according to their perceptions of taste, aroma, odor, appearance, and general acceptability. As shown in Figure 2, it was observed that the samples containing 25% whey received sensory evaluation scores close to the control sample, while the samples containing 100% whey received similar and lower scores than the other samples. It was found that there was no significant difference between the samples containing 25% whey and the control sample in terms of taste, the SW-100 sample was liked more than the AW-100 sample, and in terms of appearance and color, the samples containing 25% whey were liked more than the samples containing 100% whey but less than the control sample. The type and ratio of whey did not have a statistically significant effect on the general acceptability and aroma properties of the beverages.

### 3.4. Principle Component Analysis (PCA) and Hierarchical Cluster Analysis (HCA)

Fermented black carrot juices were grouped according to the principal component and hierarchical grouping methods in terms of analyzed properties during the fermentation process and after resting.

**Figure 3:**
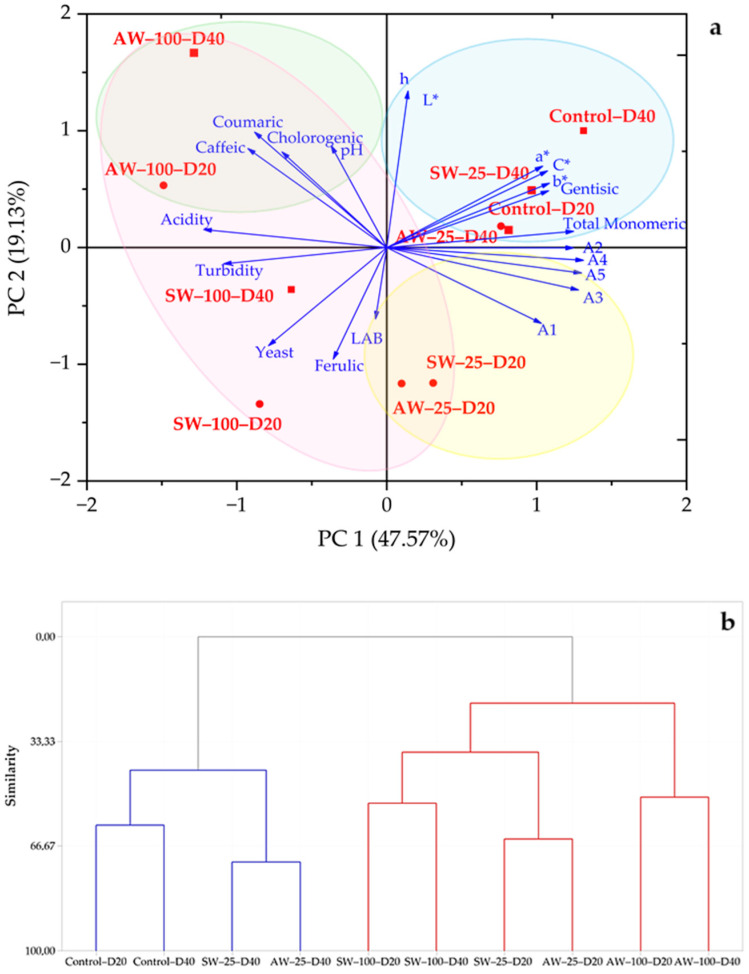
(**a**) The loading and score plot of PC1 and PC2 describing the variation among the physicochemical, microbiological, and phytochemical properties of fermented black carrot juice samples taken at different periods of fermentation; (**b**) dendrogram representation corresponding to the physicochemical, microbiological, and phytochemical properties of fermented black carrot juice samples taken at different periods of fermentation. LAB: lactic acid bacteria count; A1: cyanidin-3-xylosylglucosylgalactoside; A2: cyanidin-3-xylosylgalactoside; A3: cyanidin-3-xylosyl(sinapolyglucosyl)galactoside; A4: cyanidin-3-xylosyl(feruloylglucosyl)galactoside; A5: cyanidin-3-xylosyl(coumaroylglucosyl) galactoside; SW-25: 25% sweet whey; SW-100: 100% sweet whey; AW-25: 25% acid whey; AW-100: 100% acid whey.

Principal component analysis (PCA) was performed to examine the fermentation progress. The first five components with eigenvalues over 1 explained 94.7% of the variation. PC1 and PC2 explained 48.7% and 18.1% of the variation in the analyzed properties, respectively. Total monomeric and individual anthocyanins were positively correlated with PC1, acidity, turbidity, LAB and yeast counts, acidity, a*, b*, and C* values, and ferulic acid were positively correlated with PC4; other phenolic acids were positively correlated with PC2. L value was positively correlated with PC3, while pH and *h* values were positively correlated with PC5. PC1 can be accepted as an index of anthocyanins, PC2 phenolic acids, PC3 brightness, PC4 microbial properties, acidity and color, and PC5 pH. Figure 3a shows the loading and score plot of PC1 and PC2, which illustrates the variation among the properties of fermented black carrot juice samples. In the plot, the 40th and 20th day samples of the control and the 40th day of the samples containing 25% whey are located in the first quadrant, the 20th and 40th days of the samples containing 100% acid whey are located in the second quadrant, the 20th and 40th days of the samples containing 100% sweet whey are located in the third quadrant, and the 20th day samples of the samples containing 25% sweet whey and yogurt whey are located in the fourth quadrant.

**Figure 4:**
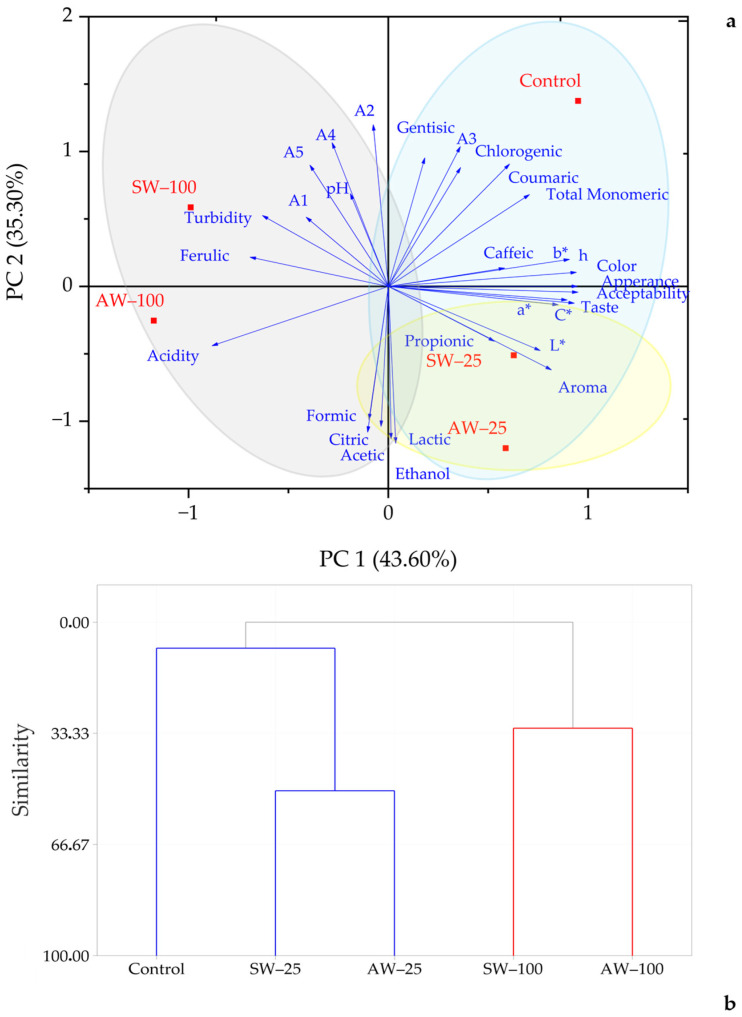
(**a**) The loading and score plot of PC1 and PC2 describing the variation among the physicochemical, sensorial, and phytochemical properties of fermented black carrot juice samples rested at 4 °C; (**b**) dendrogram representation corresponding to physicochemical, sensorial, and phytochemical properties of fermented black carrot juice samples rested at 4 °C. A1: cyanidin-3-xylosylglucosylgalactoside; A2: cyanidin-3-xylosylgalactoside; A3: cyanidin-3-xylosyl(sinapolyglucosyl)galactoside; A4: cyanidin-3-xylosyl(feruloylglucosyl)galactoside; A5: cyanidin-3-xylosyl(coumaroylglucosyl)galactoside; SW-25: 25% sweet whey; SW-100: 100% sweet whey; AW-25: 25% acid whey; AW-100: 100% acid whey.

In the samples rested at 4 °C, PC1 (42.0%) and PC2 (35.2) explained 77.1% of the variation in the analyzed properties (Figure 4a). PC1 was positively correlated with color, aroma, taste, appearance, and acceptability, and all color parameters, PC2 was positively correlated with citric, lactic, formic and acetic acids, and ethanol, and negatively correlated with gentisic, chlorogenic, coumaric, and individual anthocyanins.

Gentisic and chlorogenic acids and A1 anthocyanin were positively correlated with PC3. Figure 3b shows the loading and score plot of PC1 and PC2, which illustrates the variation among the properties of rested fermented black carrot juice samples. In the plot, the control sample is located in the first quadrant, the sample containing 100% sweet whey is located in the second quadrant, the sample containing 100% acid whey is located in the third quadrant, and the samples containing 25% sweet whey and acid whey are located in the fourth quadrant. The first component can be considered an index of color and sensory properties, and the second component an index of organic acids, individual anthocyanins, and ethanol content. The control, AW-25, and SW-25 samples were separated according to sensory and color characteristics, while AW-100 and SW-100 samples were separated according to acidity, individual anthocyanins, and organic acids.

According to the cluster analysis, the samples evaluated according to the fermentation process were divided into two main groups (Figure 3b). The first main group was divided into two subgroups as control samples and samples containing 25% whey taken on the 40th day of fermentation. In the second main group, two subgroups were formed and samples containing 100% acid whey were separated from other samples. It was observed that samples containing 25% whey taken on the 20th day were grouped within themselves, and samples containing 100% sweet whey were grouped within themselves and formed a subgroup of the second subgroup. It can be said that the control, SW-100, and AW-100 samples did not undergo any significant change in terms of the analyzed properties with the progress of fermentation, while the samples containing 25% whey were affected by the fermentation process and showed similar properties to the control samples on the 40th day of fermentation. In the samples whose fermentation was completed and ready for consumption, two main groups were obtained in the cluster analysis (Figure 4b). The first group consisted of samples containing 100% whey. The second group was divided into two subgroups, and the samples containing 25% whey formed a subgroup within itself. According to the cluster test, it can be said that ready-to-consume fermented black carrot juices containing 25% whey have similar characteristics to each other and to the control sample.

## 4. Discussion

In previous studies, the pH of fermented black carrot juice was reported to be 3.27–3.62, acidity 0.45–0.83%, turbidity 60.70–206.33 NTUs, lactic acid bacteria count 6.02–8.43 log CFU/mL, and yeast count 3.93–6.89 log CFU/mL [28,29,30,31,32,33]. The physicochemical and microbiological properties of the control sample are similar to those reported in the literature [12,30,31]. However, the addition of whey had a significant effect on the physicochemical, sensory, and microbiological attributes of fermented black carrot juice, especially with regard to anthocyanins. While lactic acid and acetic acid have been identified in previous studies on fermented black carrot juice, the presence of citric acid and propionic acid has been the subject of differing reports [28,31,34]. In this study, citric acid was not identified in the control sample, but it was identified in the samples containing whey. Considering that acidic and sweet whey contain citric acid and lactic acid [35], it is assumed that these organic acids found in fermented black carrot juice originate from whey. This hypothesis is supported by the observed increase in the concentrations of these organic acids with increasing whey content. Similarly to the results of our study, previous studies reported that the addition of whey increased the acidity and decreased the pH value of fermented tomato juice [36] and turnip juice [27].

On the 20th day of fermentation, a strong positive correlation was observed between acidity and lactic acid bacteria count (r = 0.74) and acidity and yeast count (r = 0.93). Conversely, on the 40th day of fermentation, a strong negative correlation (r = −0.80) was observed between yeast and acidity. In fermented black carrot juices, spontaneous fermentation occurs, beginning with alcoholic fermentation and subsequently progressing to lactic acid fermentation. The sourdough and carrot used for fermentation contain hetero- and homofermentative lactic acid bacteria and yeast, which work together throughout fermentation [31,37]. Metabolites produced by lactic acid bacteria inhibit yeast and Gram-negative bacteria, so homofermentative microorganisms are replaced by heterofermentative microorganisms [12]. Homofermentative microorganisms are known to produce higher levels of lactic acid, which can lead to the inhibition of less resistant microorganisms. The observed decrease in the number of LAB and yeast in the samples over the course of fermentation may be attributed to this phenomenon. The increased number of lactic acid bacteria observed on the 20th day of 100% whey-containing samples may be attributed to the action of homofermentative lactic acid bacteria transferred from the fermentation media of unpasteurized whey or to the stimulating effect of the nutritional elements present in whey on the growth of microorganisms. This is supported by the fact that total acidity and lactic acid were higher in samples containing whey. The drastic decrease in the LAB count on day 40 of fermentation can be directly related to the acidity produced. Considering the organic acid profile and ethanol levels, it is probable that both the activity of homofermentative bacteria in the whey and the biochemical composition of the whey supported the activity of heterofermentative bacteria in the fermented black carrot juice. Although the acidity was high in the sample containing 100% sweet whey, the higher pH than in the sample containing 100% sweet whey can be attributed to the free amino acids and ethanol content in the medium. It has been reported that acid whey contains a higher concentration of free amino acids and soluble peptides than sweet whey [38]. The buffering capacity of free amino acids enables them to resist pH changes [39]. It was observed that the pH and acidity levels of fermented black carrot juices produced with sweet whey were lower than those produced with acid whey. The pH of the sweet whey used was 6.07, while the pH of the acid whey was 4.40. The whey used prior to fermentation contained a certain amount of organic acids. The higher acidity of the acid whey may have influenced the acidity of the fermented product. At the same time, there may be an increase due to more lactic acid bacteria activity due to the chemical components and bacterial culture it contains. Although not statistically significant, the decrease in pH and increase in acidity of the samples during fermentation indicated that fermentation was not complete on the 20th day and microbial activity continued.

Fermented black carrot juice is a cloudy beverage. Cloudiness is caused by the addition of yeast, black carrot, and semolina to the medium prior to fermentation. These ingredients are rich in protein, hemicellulose, minerals, and polyphenolic compounds and suspension of these components in a liquid can cause haze. Furthermore, the protein and mannoprotein present in yeast have the potential to interact with the haze-forming polyphenols present in black carrot, leading to the formation of a protein–polyphenol complex. The precipitation of this complex towards the end of fermentation may have resulted in the clarification of the fermented black carrot juice. Some lactic acid bacteria strains are capable of producing polygalacturonases and/or pectin-esterase, which facilitate the conversion of pectin found in carrots into a soluble form [40].

Additionally, turbidity may have resulted from the interaction between whey proteins [41] and pectin due to the transfer of soluble pectic compounds present in carrots to the fermentation medium during fermentation, however, in low amounts. The higher protein content of the whey-containing samples may have resulted in greater protein–phenolic interactions, leading to the observed increase in turbidity. Additionally, the precipitation of this complex during fermentation and resting may have contributed to the formation of clearer fermented black carrot juices.

Yeasts adsorb anthocyanins weakly and reversibly to their cell walls at a rate of 9.4–11.9% [42]. Adsorption varies with yeast and anthocyanin type. Acyl derivatives of anthocyanins have been shown to exhibit a greater degree of adsorption than their non-acyl forms [43]. A possible reason for anthocyanin loss during fermentation may be adsorption by yeast. Monomeric anthocyanins are less stable than their polymeric forms, and during maturation in fermented beverages, monomeric anthocyanins are converted to more stable polymeric anthocyanins. The formation of polymeric pigments affects not only color but also mouthfeel.

It has been reported that at the end of alcoholic fermentation, approximately 25% of anthocyanins can undergo polymerization with a flavonoid. This rate can increase up to 40% with one year of aging. Polymerization continues at a decreasing rate until all monomeric anthocyanins are polymerized, which is completed with years of aging. It has been reported that an old red wine that is dark red in color does not contain any monomeric anthocyanins. Additionally, anthocyanins can interact with non-flavonoid phenolics to form complex pigments. Free amino acids can also undergo polymerization [16]. Mannoproteins are found in the outer layer of yeast cell walls, especially in *Saccharomyces cerevisiae* [44]. They exert a number of effects, including the reaction with anthocyanins and other phenolic compounds and the prevention of haze. Mannoproteins are composed of approximately 10–20% protein and 80% D-mannose [45]. The interaction of mannoproteins with anthocyanins is also an important factor influencing color.

In a similar manner, Güven, N., Yetim, H., and Cankurt, H. [27] reported that the anthocyanin content of turnip juices with whey was lower than that of turnip juices without whey. Consistent with the results of this study, a previous investigation of the color characteristics of whey beverages enriched with rose anthocyanin extract reported a decrease in L* and a* values and an increase in delta E values with increasing whey content [46].

It was observed that there was a significant increase in the anthocyanin amounts of the samples rested at 4 °C, especially those containing 100% whey, compared to the levels observed prior to resting. The main reason for this increase is believed to be the interaction of phenolic compounds with whey proteins.

In a study by Zang, Z., et al. [47], it was reported that the binding of chlorogenic acid to β-lactoglobulin, α-lactalbumin, and bovine serum albumin is a spontaneous reaction, mainly due to hydrophobic interaction, which increases with temperature. The authors of the study proposed that this hydrophobic interaction occurs between the benzene ring of chlorogenic acid and aliphatic, aromatic amino acids in whey proteins. The interaction affinity of whey proteins with phenolic compounds varies [48].

The binding or complexation of anthocyanins to whey proteins is spontaneous and occurs via intermolecular forces, which are mainly electrostatic and hydrophobic interactions. Studies have shown that the interaction forces of anthocyanins with various proteins include hydrogen bonding, electrostatic forces, van der Waals forces, and hydrophobic forces [49]. It has been demonstrated that anthocyanins are present in the interior regions of whey protein fractions, with the amino acid in these regions being predominantly linked to adjacent amino acid residues by hydrophobic interactions and partially by hydrogen bonding. Hydrogen bond interactions exhibit a marked increase in strength at higher temperatures, whereas hydrophilic bridge interactions demonstrate a corresponding enhancement at lower temperatures. At intermediate temperatures for a protein structure, hydrogen bonds between amino acids are stronger than those at lower temperatures [50]. The decrease in hydrophobic interactions and affinity at 4 °C may have resulted in a decrease in protein–phenolic acid and protein–anthocyanin interactions, thereby facilitating the release of phenolic and anthocyanin compounds from the complexes. The observed increase in both individual and total monomeric anthocyanins may be attributed to this situation. The reduction in the ratio of whey in the formulation resulted in a corresponding reduction in the protein ratio, leading to a reduction in the formation of the anthocyanin–protein complex. It is possible that the complex formation was more extensive in samples containing a higher percentage of whey. The observed increase in the amounts of cyanidin-3-xylosylglucosylgalactoside, cyanidin-3-xylosylgalactoside, and cyanidin-3-xylosyl(feruloylglucosyl)galactoside in anthocyanins, particularly in fermented black carrot juices containing 100% whey after resting, can be attributed to the release of anthocyanins from the formed protein–anthocyanin complex into a free form.

It has been reported that the interaction of anthocyanins with whey proteins has the effect of improving the stability and nutritional and functional properties of anthocyanins, increasing their bioavailability, antioxidant capacity, digestive, absorptive, and emulsifying properties [49]. Khalifa, I., et al. [51] reported that whey protein has a co-pigmentation effect on anthocyanins and increases their stability. Furthermore, they noted that anthocyanins encapsulated with whey protein are pale in powder form but become more colored when reconstituted. In vitro simulated digestion experiments have demonstrated that the degradation of anthocyanins is inhibited as a result of the interaction of anthocyanins with whey protein [47].

A comparable situation was observed with chlorogenic acid, the primary phenolic compound present in fermented black carrot juice. Polyphenols interact with proteins by non-covalent conjugation via -OH groups in acidic environments, and these are less stable and reversible than protein–phenolic [52] covalent conjugations, which mostly occur in basic environments. The interaction between phenolic compounds and proteins is influenced by several factors, including pH, temperature, the presence of other compounds in the environment, the structure of the protein, and the molecular weight of the phenolic compound. As the molecular weight of the protein increases, the binding affinity for phenolics tends to increase [53]. Additionally, temperature plays a crucial role in determining the ability of phenolics to bind to proteins [44,54].

The acidity of commercially produced fermented black carrot juices is approximately 0.6–0.8%. In this study, the acidity of fermented black juices containing 100% whey was found to exceed 1.0%. The high acidity of the 100% whey fermented black carrot juice samples was less appreciated sensorially in terms of taste, while the lower anthocyanin content resulted in the sample containing 100% whey being less favorable in terms of color and appearance.

## 5. Conclusions

In this study, the changes in phenolic compounds, microbiological, sensory, and physicochemical properties of fermented black carrot juice during the fermentation process and after the cold resting process in the cases of using sweet and acid whey in the formulation were revealed. In general, whey accelerated fermentation, improved the microbiological properties of the beverage, and created a difference in phenolic compounds by forming a reversible complex. During the fermentation process, an increase in turbidity and acidity was observed as the amount of whey in the beverage increased. Conversely, a decrease in pH, redness, yellowness, color saturation, and individual anthocyanin content was observed. The beverages produced with acid whey were more acidic and cloudy, while those produced with sweet whey had a lower pH and higher anthocyanin content. The whey type had no significant effect on color parameters.

In beverages prepared for consumption after a resting period, an increase in acidity and a decrease in pH were observed with increasing whey content. No significant differences in color and turbidity were observed. Sweet whey-containing beverages had lower pH values and higher anthocyanin content than acid whey-containing beverages. Classification by PCA and HCA methods showed that the samples containing 25% whey were grouped with the control sample and therefore the samples containing 25% whey were similar to the control sample. The results of the study show that both acid and sweet whey can be used in the production of fermented black carrot juice. However, instead of using whey alone, it may be more appropriate to use a mixture of whey and water in certain proportions. Although the beverage obtained as a result of the research is a waste utilization product, the inclusion of a more expensive raw material than water in the formulation will increase the cost of the fermented carrot juice to be produced, but the fact that a product with increased functionality is obtained, enriched in terms of protein and mineral content, will increase consumer acceptance. It is recommended that 25% whey be used in the formulation and that the product be rested at 4 °C before consumption. This research will serve as the basis for further studies investigating the aroma characteristics and microbial dynamics of whey-based beverages.

## Figures and Tables

**Table 1:** Some physicochemical properties of fermented black carrot juice on 20th and 40th days of fermentation.

Samples	FP (Day)	pH	Titratable Acidity (g LA/100 mL)	Turbidity (NTU)	TMA (mg CGE/L)
Control	20	3.49 ± 0.04	0.349 ± 0.025	188.50 ± 6.50 ^d^	63.62 ± 2.50
	40	3.46 ± 0.04	0.460 ± 0.042	120.50 ± 5.50 ^ef^	58.61 ± 0.67
SW-25 *	20	3.12 ± 0.01	0.855 ± 0.015	285.00 ± 13.00 ^c^	38.16 ± 1.50
	40	3.09 ± 0.02	0.889 ± 0.031	75.60 ± 4.40 ^fg^	38.28 ± 0.21
SW-100	20	3.29 ± 0.02	1.147 ± 0.073	294.00 ± 16.00 ^c^	11.86 ± 0.33
	40	3.22 ± 0.03	1.215 ± 0.114	153.00 ± 4.00 ^de^	12.69 ± 0.25
AW-25	20	3.26 ± 0.03	0.801 ± 0.047	375.00 ± 14.00 ^b^	34.11 ± 0.21
	40	3.22 ± 0.01	0.938 ± 0.052	50.40 ± 1.79 ^g^	35.49 ± 0.58
AW-100	20	3.47 ± 0.01	1.426 ± 0.088	513.00 ± 7.00 ^a^	12.15 ± 1.04
	40	3.45 ± 0.04	1.404 ± 0.104	377.00 ± 12.00 ^b^	11.06 ± 0.71

* SW—sweet whey; AW—acid whey; FP—fermentation period; NTU—Nephelometric Turbidity Unit; LA—lactic acid; TMA—total monomeric anthocyanin; 25 and 100 next to the abbreviations indicate the whey ratio as a water substitute. Different letters in the same column indicate the difference between means and there is no difference between the samples due to the fermentation periods in the properties that do not include letters.

**Table 2:** Some physicochemical properties of the fermented black carrot juice rested at 4 °C for 2 days.

Samples	pH	Titratable Acidity (g LA/100 mL)	Turbidity (NTU)	TMA (mg CGE/L)
Control	3.46 ± 0.03 ^a^	0.490 ± 0.020 ^c^	63.30 ± 11.03	65.13 ± 0.88 ^a^
SW-25 *	3.08 ± 0.06 ^c^	0.860 ± 0.040 ^b^	54.20 ± 3.50	59.91 ± 0.35 ^b^
SW-100	3.24 ± 0.02 ^bc^	1.160 ± 0.010 ^a^	61.70 ± 8.50	52.46 ± 0.43 ^c^
AW-25	3.22 ± 0.01 ^c^	0.880 ± 0.020 ^b^	54.05 ± 4.35	53.16 ± 0.85 ^c^
AW-100	3.43 ± 0.03 ^ab^	1.370 ± 0.070 ^a^	71.60 ± 2.40	52.81 ± 0.54 ^c^

* SW—sweet whey; AW—acid whey; NTU—Nephelometric Turbidity Unit; LA—lactic acid; TMA—total monomeric anthocyanin; 25 and 100 next to the abbreviations indicate the whey ratio as a water substitute. Different letters in the same column indicate the difference between means and there is no difference between the samples due to resting in the properties that do not include letters.

**Table 3:** Color parameters of fermented black carrot juice on 20th and 40th days of fermentation.

Samples	FP (Day)	L*	a*	b*	C*	*h*
Control	20	17.60 ± 1.17 ^de^	41.22 ± 0.86 ^b^	27.85 ± 1.45 ^b^	49.75 ± 1.52 ^b^	34.02 ± 0.83 ^abc^
	40	29.46 ± 4.05 ^bc^	53.83 ± 5.59 ^a^	40.37 ± 4.78 ^a^	67.29 ± 7.34 ^a^	36.83 ± 0.40 ^ab^
SW-25 *	20	10.32 ± 0.54 ^e^	31.37 ± 0.63 ^bc^	16.61 ± 0.51 ^c^	35.50 ± 0.50 ^bc^	27.90 ± 0.92 ^de^
	40	33.74 ± 0.39 ^b^	55.56 ± 0.44 ^a^	49.70 ± 0.70 ^a^	69.70 ± 0.58 ^a^	37.14 ± 0.77 ^a^
SW-100	20	9.11 ± 1.09 ^e^	28.22 ± 1.88 ^c^	13.38 ± 1.53 ^c^	31.23 ± 1.34 ^c^	25.37 ± 0.75 ^ef^
	40	23.76 ± 2.18 ^cd^	28.70 ± 2.09 ^c^	13.51 ± 2.19 ^c^	31.72 ± 1.92 ^c^	34.20 ± 0.83 ^abc^
AW-25	20	16.32 ± 0.69 ^de^	36.47 ± 0.62 ^bc^	23.92 ± 0.58 ^bc^	43.61 ± 0.52 ^bc^	33.26 ± 0.47 ^bc^
	40	37.99 ± 1.83 ^ab^	59.29 ± 1.16 ^a^	40.29 ± 1.11 ^a^	71.69 ± 1.25 ^a^	34.20 ± 0.52 ^abc^
AW-100	20	18.36 ± 1.21 ^de^	36.99 ± 1.19 ^bc^	22.58 ± 1.34 ^bc^	43.34 ± 1.21 ^bc^	31.41 ± 0.64 ^cd^
	40	45.85 ± 1.44 ^a^	32.51 ± 1.52 ^bc^	13.03 ± 1.47 ^c^	35.03 ± 1.57 ^c^	21.85 ± 0.36 ^f^

* SW—sweet whey; AW—acid whey; FP—fermentation period; 25 and 100 next to the abbreviations indicate the whey ratio as a water substitute. Different letters in the same column indicate the difference between means.

**Table 4:** Color parameters of fermented black carrot juice rested at 4 °C for 2 days.

Samples	L*	a*	b*	C*	*h*
Control	38.65 ± 1.53	64.33 ± 2.47	49.77 ± 9.84	81.34 ± 7.71	37.73 ± 4.89
SW-25 *	38.00 ± 0.52	62.43 ± 3.69	52.81 ± 3.85	81.77 ± 3.59	40.23 ± 4.69
SW-100	34.19 ± 0.62	56.89 ± 3.29	43.96 ± 5.80	71.89 ± 2.85	37.69 ± 1.26
AW-25	40.05 ± 1.11	63.55 ± 2.06	48.50 ± 4.87	79.94 ± 7.33	37.35 ± 5.21
AW-100	36.75 ± 8.26	60.00 ± 6.66	41.56 ± 5.73	72.98 ± 2.96	34.71 ± 3.54

* SW—sweet whey; AW—acid whey; 25 and 100 next to the abbreviations indicate the whey ratio as a water substitute. There is no difference between the samples due to resting in the properties that do not include letters.

**Table 5:** Phenolics acids (mg/L) detected in fermented black carrot juice on 20th and 40th days of fermentation.

Samples	FP (Day)	Gentisic	Chlorogenic	Caffeic	*p*-Coumaric	Ferulic
Control	20	1.04 ± 0.12	59.56 ± 2.44	4.49 ± 0.52	0.66 ± 0.05	nd
	40	1.10 ± 0.02	49.22 ± 1.45	3.97 ± 0.19	0.64 ± 0.07	nd
SW-25	20	0.53 ± 0.06	64.90 ± 0.94	4.61 ± 1.06	0.54 ± 0.07	0.08 ± 0.04
	40	0.54 ± 0.09	57.43 ± 1.00	4.31 ± 0.16	0.52 ± 0.08	0.07 ± 0.01
SW-100	20	0.17 ± 0.10	46.37 ± 1.34	3.95 ± 0.61	0.56 ± 0.12	0.19 ± 0.02
	40	0.18 ± 0.03	44.59 ± 0.50	3.63 ± 0.37	0.51 ± 0.02	0.14 ± 0.02
AW-25	20	0.38 ± 0.08	44.14 ± 1.86	2.15 ± 0.60	0.45 ± 0.14	0.78 ± 0.07
	40	0.35 ± 0.04	44.59 ± 0.53	1.79 ± 0.21	0.42 ± 0.09	0.81 ± 0.08
AW-100	20	0.29 ± 0.03	81.71 ± 0.57	9.64 ± 0.83	1.02 ± 0.48	0.08 ± 0.00
	40	0.31 ± 0.08	77.02 ± 1.14	8.03 ± 0.47	0.94 ± 0.17	0.07 ± 0.03

nd: nondetectable. SW—sweet whey; AW—acid whey; FP—fermentation period; 25 and 100 next to the abbreviations indicate the whey ratio as a water substitute. There is no difference between the samples due to the fermentation periods in the properties that do not include letters.

**Table 6:** Phenolics acids (mg/L) detected in fermented black carrot juice rested at 4 °C for 2 days.

Samples	Gentisic	Chlorogenic	Caffeic	*p*-Coumaric	Ferulic
Control	2.11 ± 0.18 ^a^	70.33 ± 1.70 ^a^	4.43 ± 0.60	0.91 ± 0.12	0.11 ± 0.08
SW-25	0.70 ± 0.11 ^b^	63.66 ± 1.23 ^a^	3.83 ± 0.24	0.64 ± 0.15	0.01 ± 0.00
SW-100	1.98 ± 0.02 ^a^	67.09 ± 1.34 ^a^	4.31 ± 0.19	0.64 ± 0.11	0.12 ± 0.06
AW-25	0.99 ± 0.07 ^b^	50.19 ± 1.84 ^b^	4.70 ± 0.31	0.55 ± 0.07	0.08 ± 0.02
AW-100	0.41 ± 0.15 ^b^	45.24 ± 0.85 ^b^	2.75 ± 0.38	0.42 ± 0.15	0.25 ± 0.07

SW—sweet whey; AW—acid whey; 25 and 100 next to the abbreviations indicate the whey ratio as a water substitute. Different letters in the same column indicate the difference between means and there is no difference between the samples due to resting in the properties that do not include letters.

**Table 7:** Anthocyanins (mg/L) of fermented black carrot juice on 20th and 40th days of fermentation.

Samples	FP (Day)	A1	A2	A3	A4	A5
Control	20	1.70 ± 0.02	24.69 ± 1.43	4.28 ± 0.69	27.31 ± 1.19	11.00 ± 0.78
	40	1.54 ± 0.16	23.28 ± 0.75	5.14 ± 0.11	25.81 ± 0.29	12.65 ± 0.47
SW-25	20	3.43 ± 0.31	19.24 ± 0.79	4.73 ± 0.36	22.61 ± 4.37	13.14 ± 0.27
	40	2.75 ± 0.95	17.18 ± 1.58	4.38 ± 0.41	22.84 ± 0.31	11.95 ± 0.45
SW-100	20	0.92 ± 0.18	2.90 ± 0.13	1.08 ± 0.04	12.33 ± 1.07	6.01 ± 0.70
	40	0.80 ± 0.17	2.76 ± 0.28	0.98 ± 0.26	10.28 ± 1.37	5.51 ± 0.59
AW-25	20	2.43 ± 0.14	11.01 ± 1.03	4.71 ± 0.30	17.10 ± 0.89	7.93 ± 0.58
	40	2.89 ± 0.90	10.38 ± 0.67	4.47 ± 0.51	18.98 ± 0.12	9.83 ± 0.52
AW-100	20	0.35 ± 0.07	0.94 ± 0.09	0.58 ± 0.11	7.46 ± 0.63	3.32 ± 0.71
	40	0.32 ± 0.13	0.97 ± 0.15	0.56 ± 0.32	7.83 ± 0.37	3.79 ± 0.40

A1—cyanidin-3-xylosylglucosylgalactoside; A2—cyanidin-3-xylosylgalactoside; A3—cyanidin-3-xylosyl(sinapolyglucosyl)galactoside; A4—cyanidin-3-xylosyl(feruloylglucosyl)galactoside; A5—cyanidin-3-xylosyl(coumaroylglucosyl)galactoside. SW—sweet whey; AW—acid whey; FP—fermentation period; 25 and 100 next to the abbreviations indicate the whey ratio as a water substitute. There is no difference between the samples due to the fermentation periods in the properties that do not include letters.

**Table 8:** Anthocyanins (mg/L) of fermented black carrot juice rested at 4 °C for 2 days.

Sample	A1	A2	A3	A4	A5
Control	6.30 ± 0.65	27.35 ± 0.73 ^a^	7.82 ± 0.33 ^a^	32.91 ± 0.07 ^a^	16.08 ± 1.32 ^ab^
SW-25	7.23 ± 0.79	15.82 ± 0.63 ^bc^	5.06 ± 0.64 ^b^	23.39 ± 1.01 ^bc^	11.71 ± 0.41 ^b^
SW-100	8.97 ± 0.04	24.44 ± 0.97 ^a^	4.55 ± 0.46 ^b^	36.13 ± 0.30 ^a^	20.01 ± 1.09 ^a^
AW-25	5.25 ± 0.93	11.00 ± 0.90 ^c^	3.27 ± 0.44 ^b^	21.55 ± 0.75 ^c^	11.66 ± 0.74 ^b^
AW-100	6.10 ± 0.82	17.26 ± 1.15 ^b^	4.67 ± 0.29 ^b^	25.76 ± 0.34 ^b^	13.21 ± 0.63 ^b^

A1—cyanidin-3-xylosylglucosylgalactoside; A2—cyanidin-3-xylosylgalactoside; A3—cyanidin-3-xylosyl(sinapolyglucosyl)galactoside; A4—cyanidin-3-xylosyl(feruloylglucosyl)galactoside; A5—cyanidin-3-xylosyl(coumaroylglucosyl)galactoside. SW—sweet whey; AW—acid whey; 25 and 100 next to the abbreviations indicate the whey ratio as a water substitute. Different letters in the same column indicate the difference between means and there is no difference between the samples due to resting in the properties that do not include letters.

**Table 9:** Organic acids (g/100 mL) and ethanol contents (g/100 mL) of fermented black carrot juice.

Organic Acids and Ethanol	Control	SW-25	SW-100	AW-25	AW-100
Citric	nd	0.194 ± 0.014 ^a^	0.266 ± 0.054 ^a^	0.163 ± 0.027 ^ab^	0.286 ± 0.034 ^a^
Lactic	0.264 ± 0.026 ^d^	0.665 ± 0.015 ^c^	0.892 ± 0.029 ^b^	0.725 ± 0.035 ^bc^	1.290 ± 0.040 ^a^
Formic	0.003 ± 0.001 ^c^	0.028 ± 0.003 ^ab^	0.038 ± 0.002 ^a^	0.021 ± 0.002 ^b^	0.034 ± 0.002 ^a^
Propionic	0.087 ± 0.004 ^b^	0.039 ± 0.002 ^d^	0.056 ± 0.004 ^cd^	0.069 ± 0.002 ^bc^	0.113 ± 0.007 ^a^
Acetic	0.031 ± 0.006 ^b^	0.106 ± 0.014 ^ab^	0.136 ± 0.015 ^a^	0.084 ± 0.011 ^ab^	0.139 ± 0.021 ^a^
Ethanol	0.438 ± 0.048 ^d^	0.537 ± 0.043 ^cd^	0.728 ± 0.024 ^bc^	0.854 ± 0.034 ^b^	1.158 ± 0.061 ^a^

nd: non-detectable. SW—sweet whey; AW—acid whey; 25 and 100 next to the abbreviations indicate the whey ratio as a water substitute. Different letters in the same row indicate the difference between averages and there is no difference between the samples due to resting in the properties that do not include letters.

**Table 10:** Yeast and lactic acid bacteria (LAB) count of fermented black carrot juice on 20th and 40th days of fermentation.

Samples	Fermentation Period (Day)	Lactic Acid Bacteria (Log CFU/mL)	Yeast Counts (Log CFU/mL)
Control	20	6.43 ± 0.08 ^c^	6.70 ± 0.15
	40	4.08 ± 0.06 ^e^	4.07 ± 0.07
SW-25	20	7.53 ± 0.13 ^b^	7.37 ± 0.07
	40	5.44 ± 0.05 ^d^	4.44 ± 0.17
SW-100	20	8.04 ± 0.06 ^a^	8.10 ± 0.08
	40	<1	4.18 ± 0.09
AW-25	20	6.43 ± 0.10 ^c^	7.50 ± 0.08
	40	4.42 ± 0.09 ^e^	4.21 ± 0.22
AW-100	20	8.08 ± 0.07 ^a^	8.11 ± 0.09
	40	3.43 ± 0.07 ^f^	4.17 ± 0.11

SW—sweet whey; AW—acid whey; CFU—Colony Forming Unit; 25 and 100 next to the abbreviations indicate the whey ratio as a water substitute. Different letters in the same column indicate the difference between means and there is no difference between the samples due to the fermentation periods in the properties that do not include letters.

## Data Availability

The original contributions presented in the study are included in the article, further inquiries can be directed to the corresponding author.

## Supplementary Materials

The following supporting information can be downloaded at https://www.mdpi.com/article/10.3390/foods14020218/s1. Table S1: *p* values of two-sample t test results for physicochemical and color values of the samples (fermentation times: 40th day); Table S2: *p* values of two-sample t test results for the phenolic acids and anthocyanins values of the samples (fermentation times: 40th day).
