# Supplementary material for: Valorization of Dairy By-Products, Sweet Whey, and Acid Whey, in the Production of Fermented Black Carrot Juice: A Comparative Study of the Phytochemical, Physicochemical, Microbiological, and Sensorial Aspects
Source: Foods. 2025 Jan 12;14(2):218. doi: 10.3390/foods14020218 (PMC11765452; doi:10.3390/foods14020218)
Supplement: Supplementary file 1 [file foods-14-00218-s001.zip › foods-3363671-supplementary.pdf]

Table S1. *p* values of two-sample t test results for physicochemical and color values of the samples (fermentation times: 40th day)

**Samples**	**pH**	**Acidity**	**Turbidity**	**TMA**	***L****	***a****	***b****	**C***	***h***
Control<br>Control−F **	1.00	0.635	0.105	0.107	0.280	0.336	0.351	0.413	0.884
SW−25<br>SW−25−F	0.90	0.669	0.164	**0.012**	0.096	0.316	0.572	0.186	0.633
SW−100<br>SW−100−F	0.535	0.715	0.065	**0.008**	0.136	0.085	0.128	0.054	0.260
AW−25<br>AW−25−F	1.00	0.487	0.580	**0.037**	0.512	0.323	0.348	0.467	0.655
AW−100<br>AW−100−F	0.758	0.831	**0.025**	**0.014**	0.474	0.155	0.130	0.056	0.172

** F indicates 2 days of resting at 4 ^{o}C at the end of 40 days of fermentation; TMA−total monomeric anthocyanin

Table S2. . p values of two-sample t test results for the phenolic acids and anthocyanins values of the samples (fermentation times: 40th day)

**Samples**	**Gentisic**	**Chlorogenic**	**Caffeic**	**Ferulic**	***p*-Coumaric**	**A1**	**A2**	**A3**	**A4**	**A5**
Control<br>Control−F*	0.114	0.067	0.595	-	0.302	0.089	0.161	0.083	**0.026**	0.247
SW−25<br>SW−25−F	0.491	0.159	0.346	0.587	0.101	0.172	0.573	0.539	0.692	0.769
SW−100<br>SW−100−F	**0.013**	**0.040**	0.353	0.454	0.821	**0.014**	**0.030**	0.093	**0.035**	0.054
AW−25<br>AW−25−F	0.077	0.210	0.082	0.475	0.075	0.319	0.675	0.324	0.182	0.294
AW−100<br>AW−100−F	0.665	**0.028**	0.073	0.261	0.231	0.090	**0.045**	0.067	**0.018**	0.050

*F indicates 2 days of resting at 4 °C at the end of 40 days of fermentation; A1—cyanidin-3-xylosylglucosylgalactoside; A2—cyanidin-3-xylosylgalactoside; A3—cyanidin-3-xylosyl(sinapolyglucosyl)galactoside; A4—cyanidin-3-xylosyl(feruloylglucosyl)galactoside; A5—cyanidin-3-xylosyl(coumaroylglucosyl)galactoside.
